# Structure guided functional analysis of the S. cerevisiae Mre11 complex

**DOI:** 10.21203/rs.3.rs-5390974/v1

**Published:** 2024-12-09

**Authors:** John Petrini, Marcel Hohl, You Yu, Vitaly Kuryavyi, Dinshaw Patel

**Affiliations:** Memorial Sloan Kettering Cancer Center; Memorial Sloan-Kettering; Zhejiang University School of Medicine; MSKCC; Memorial Sloan Kettering Cancer Center

**Keywords:** DNA repair, Rad50, Mre11 complex, Cryo-EM, hook, double strand break, coiled coil

## Abstract

The Mre11 complex comprises Mre11, Rad50 and Nbs1 (Xrs2 in *S. cerevisiae*). The core components, Mre11 and Rad50 are highly conserved, with readily identifiable orthologs in all clades of life, whereas Nbs1/Xrs2 are present only in eukaryotes. In eukaryotes, the complex is integral to the DNA damage response, acting in DNA double strand break (DSB) detection and repair, and the activation of DNA damage signaling. We present here a 3.2 Å cryo-EM structure of the *S. cerevisiae* Mre11-Rad50 complex with bound dsDNA. The structure provided a foundation for detailed mutational analyses regarding homo and heterotypic protein interfaces, as well as DNA binding properties of Rad50. We define several conserved residues in Rad50 and Mre11 that are critical to complex assembly as well as for DNA binding. In addition, the data reveal that the Rad50 coiled coil domain influences ATP hydrolysis over long distances.

## INTRODUCTION

The Mre11 complex is integral to the DNA damage response (DDR). The complex promotes DNA double strand break (DSB) repair and in eukaryotes, it activates the Tel1/ATM kinase to initiate DNA damage signaling. The complex is comprised of two molecules each of Mre11 and Rad50, and one molecule of Nbs1 (Xrs2 in budding yeast)^1^. Orthologs of Mre11 and Rad50 are found in all clades of life, whereas Nbs1/Xrs2 is exclusive to Eukarya. Mre11 and Rad50 (MR) specify enzymatic functions (nuclease and ATPase, respectively), whereas Nbs1/Xrs2 is neither an enzyme, nor does it bind DNA. Recent studies demonstrate that the essential function of Nbs1/Xrs2 is to facilitate proper assembly of the complex, which in turn promotes nuclear localization^2,3^. Nevertheless, there is some evidence for Nbs1/Xrs2 independent functions of the MR complex in *S. cerevisiae*^4^.

The Mre11 complex is a distant member of the Structure Maintenance of Chromosomes (SMC) family of proteins; however, there are two important distinctions between the Mre11 complex and classical SMC proteins. First, whereas SMC proteins interact via a hinge domain that lies within their respective coiled coil regions, Rad50 dimerizes in the analogous region through a zinc binding domain assembled from two Rad50 protomers termed the Rad50 Zn hook domain. Second, the complex does not contain kleisin subunits, which are found in SMC complexes such as cohesin, condensin and Smc5/6 associated with the Walker A and B ATPase domains^5^. Instead, the Rad50 Walker A and B domains from each of the two Rad50 protomers associate with each other in part by binding two ATP molecules^6^.

The Mre11 complex appears to function in two forms. In the ATP-bound closed form, the Rad50 Walker A and B domains from each of the two Rad50 protomers associate with each other, with the adjacent coiled coils adopting a rod-shaped structure. This form appears to primarily influence Tel1/ATM activation and non-homologous endjoining^7–9^. Upon ATP hydrolysis, the Mre11 complex assumes an open form in which the two ATP binding domains rotate outward, such that the Mre11 dimer interface, in which the nuclease active sites reside, become accessible. Accordingly, the open form has been implicated in Mre11 complex-mediated DSB end resection and repair by homologous recombination^6^.

In contrast, recent cryo-EM analysis of the SbcCD complex, the *E. coli* ortholog of the eukaryotic MR complex, presents a different view. The structures obtained reveal that in the absence of DNA, the Rad50 coiled coils are in an open, ring-shaped form with the Mre11 dimer underneath the Rad50 ATPase domains. This form senses a DNA end, which subsequently induces “zippering” of the coiled coils into a closed rod-shaped form. Zippering of the coiled coils is accompanied by SbcD (Mre11) relocalizing to the side of Rad50, where it binds the DNA end and creates a nuclease channel in which DNA ends or hairpin structures are processed prior to homology directed DSB repair^10,11^. This raises the possibility that the structural transitions themselves, as opposed to the open and closed endpoints, may be functionally important.

The mechanisms underlying the transition from the closed to open forms of the Mre11 complex require further investigation. In previous studies, we determined the crystal structure of a 182-aa fragment spanning the human Rad50 Zn hook domain. Those data demonstrate that the apex of the Rad50 coiled coils form a rod-shaped structure that is unlikely to come apart during the transition from the closed to open forms of the complex. In addition to the zinc-binding interface situated at the apex, the adjacent coiled coils form a second interface that is roughly equivalent in size to the zinc binding interface (*ca*., 900 Å^2^). This coiled coil interface is situated between the apical zinc hook interface and a highly flexible five-residue “hinge” loop (CGSQD)^12^, suggesting that this flexible region permits mobility of this region of the coiled coils. Given that the apical region of Rad50 is rigid and stable, this hinge domain, and possibly additional regions of coiled coil flexibility, are presumably required for the opening of the closed complex.

The available evidence suggests at least two modes of DNA binding by the Mre11 complex that are specific to the closed and open forms of the complex. We used cryo-EM to examine the globular domain and the proximal coiled coil domain of the *S. cerevisiae* MR complex to gain insights regarding DNA engagement, the interfaces that mediate assembly of the complex, and to probe the mechanism of ATM/Tel1 activation. We report herein a 3.2 Å structure of the closed form of the MR complex with bound dsDNA. Reminiscent of the *E. coli*. structure of the MR-dsDNA complex^10^, the coiled coils encircle the DNA and come together above it. Mre11 does not make contacts with the dsDNA, suggesting that this DNA bound structure represents a precursor to the open form of the complex, as predicted previously^13^. A recently reported structure of the *C. thermophilum* MRN complex bound to ATPγS revealed a similar rod-shaped structure that did not include DNA^11^.

## RESULTS

**Figure 1:**
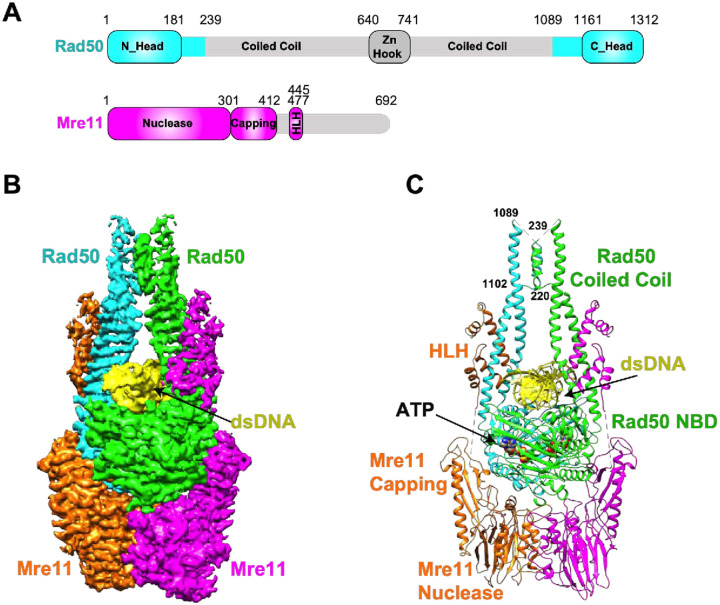
Cryo-EM structures of *S. cerevisiae* MR-DNA complex. **(A)** Domain architecture of *S. cerevisiae* Rad50 and Mre11. **(B, C)** The 3.2 Å cryo-EM structure of MR-DNA complex in electron density (panel B) and ribbon (panel C) representations. The symmetry related Rad50 subunits are colored in cyan and green, the symmetry related Mre11 subunits are colored in orange and magenta, and the bound dsDNA is in yellow. The bound ATPs are shown in space-filling representation in panel C.

The domain organization of the Mre11 and Rad50 protomers is outlined in Fig. 1A. Rad50 contains a tripartite filamentous architecture in which the N- and C-terminal Walker A and B domains are separated by two long arms (*ca*. 400-aa) of coiled coil segments. The N-terminal arm terminates at a 100-aa domain comprising the zinc hook domain, which constitutes a Zn-dependent dimerization interface. The C-terminal coiled coil arm begins at the zinc hook and extends to the Walker B domain. The Rad50 protein folds at the hook domain such that the N- and C-arms of the coiled coils are in an antiparallel configuration and the Walker A and B domains interface to form part of the Mre11 complex globular domain. Mre11 has an N-terminal Mn^2+^ dependent phosphoesterase (nuclease) domain, access to which is regulated by a capping domain. There is a helix-loop-helix (HLH) motif C-terminal to the capping domain that appears to bind the Rad50 coil coils proximal to the globular domain, while the remainder of the C-terminus is largely disordered^9^. Nbs1 (Xrs2 in *S. cerevisiae*) is found only in Eukarya. It has N-terminal FHA and BRCT domains and a highly conserved ten amino acid motif (RKNFKTFVKV in *S. cerevisiae*) that is necessary and sufficient for interaction with the Mre11 dimer. The remaining protein segments appear to be disordered^2,14^.

We purified the Mre11-Rad50-Xrs2 (MRX) complex from *S. cerevisiae* to carry out structural studies. We were unable to obtain useable MRX particles with or without DNA and crosslinking. To minimize the spatial dynamics of the complex, and to avoid the confounding effects of Xrs2, we next purified the Mre11-Rad50 (MR) complex, in which the E1235 residue of Rad50 was mutated to Q (R^EQ^) to prevent ATP hydrolysis^9^, which likely drives the transition from the closed to the open form of the complex^13 15^.

### Cryo-EM structure of the MR-dsDNA complex

MR^EQ^, with Mre11 containing a C-terminal 1xFLAG tag, was purified from yeast extracts by sequential affinity purification to FLAG-Agarose, Heparin-Sepharose and peak fractionation by gel filtration chromatography (Figure S1A) to obtain highly pure MR^EQ^ (Figure S1B). MR^EQ^ was incubated with a 83-mer double stranded DNA (dsDNA) in the presence of ATP, MgCl_2_ and MnCl_2_ prior to making the cryo-EM grids. We also made cryo-EM grids without dsDNA; however, we did not get enough particles for 2D classification.

The structure of the MR-dsDNA complex at an overall resolution of 3.2 Å (cryo-EM work flow listed in Figure S2, FSC curve in Figure S3A, angular distribution plot in Figure S3B, and statistics in Table S1) is shown in density and ribbon representations in Figs. 1B and 1C, respectively. The central component of the complex spanning the head segment of Rad50 and the nuclease with capping domains of Mre11 can be monitored at the highest resolution (Figure S3C), as reflected in the tracing of side chains of these segments (Figures S3D–S3F). The correct density tracing of these segments was facilitated by incorporation of secondary structure elements derived from AlphaFold2 and SCHRÖDINGER program-based homology modeling. The head-proximal coiled coil arms of Rad50 and the HLH domain of Mre11 can be monitored at medium resolution (3.5 to 4.0 Å; Figure S3C), with these segments suitable for structural rigid body fit and refinement. By contrast, traceable coiled coil arm segments of Rad50 (residues 220–239 and 1089–1102) are observed at lower resolution, while the remainder of the coiled coil arm and Zn hook segments (residues 239–1089) of Rad50, the segment (residues 413–441) connecting the capping and HLH domains of Mre11 and the C-terminus of Mre11 (residues 508–692), are flexible and cannot be traced in the complex (Figs. 1B, 1C and S3C). Notably, we can also monitor about two turns of bound dsDNA in the MR-DNA complex (Figs. 1B and 1C).

### Rad50 head domain alignment and Mre11 nuclease pocket

The pair of ATPase domains of Rad50 in the *S. cerevisiae* MR (data not shown) and MR-dsDNA complex (Figure S3G) in this study both adopt an “engaged” alignment, following comparison with the “engaged” alignment observed in the *S. cerevisiae* Cohesin (PDB: 6ZZ6) versus the “juxtaposed” alignment observed in the *S. cerevisiae* Condensin (PDB: 6YVU).

Both the catalytic residues and divalent cations in the Mre11 nuclease active site of the cryo-EM structure of the *S. cerevisiae* MR-dsDNA complex reported in this study (Figure S3H, in color) superpose well with those of the x-ray structure of the *P. furiosus* Mre11 (PDB: 1S8E; Figure S3H, in silver).

### Protein-protein contacts involving Mre11 capping domain with Rad50 head domain

**Figure 2:**
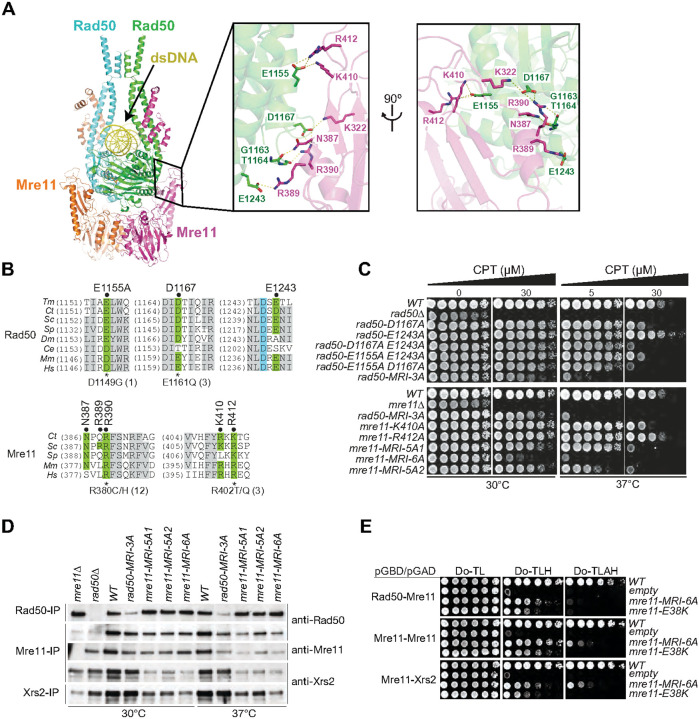
Mutations in globular domain residues of Mre11-Rad50 partially destabilize complex formation and interaction with Xrs2. **(A)** The boxed panels show two views rotated by 90° of protein-protein interactions between the Mre11 capping domain and Rad50 head domain in the MR-dsDNA complex. Conserved Rad50 E1155, D1167 and E1243 residues and Mre11 N387, R389, R390, K410 and R412 residues are labeled in the boxed segments. **(B)** Evolutionary conservation of Rad50 (top) and Mre11 (bottom) residues mediating protein-protein interactions. *Tm, Thermotoga maritime; Ct, Chaetomium thermophilum; Sc, Saccharomyces cerevisiae; Sp, Schizosaccharomyces pombe; Dm, Drosophila melanogaster; Ce, Caenorhabditis elegans; Mm, Mus musculus; Hs, Homo sapiens*. Rad50 and Mre11 tumor alleles and their number of instances (in brackets) are depicted below the alignments. **(C)** CPT-survival of *rad50* (top) and *mre11* (bottom) mutants in the Mre11-Rad50 interaction interface. Plates were either incubated at 30°C (left panel) or 37°C (right panel). The following abbreviations were used: *rad50-MRI-3A* (*rad50-E1155A D1167A E1243A*); *mre11-MRI-5A1* (*K322A N387A R389A R390A R412A*); *mre11-MRI-5A2* (*K322A N387A R389A R390A K410A*); *mre11-MRI-6A* (*K322A N387A R389A R390A K410A R412A*). **(D)** Assessment of the Mre11-Rad50 and Mre11-Xrs2 interaction by co-immunoprecipitation. Cells were either grown at 30°C (left panel) or 37°C (right panel) and Rad50, Mre11 or Xrs2 were immunoprecipitated from the prepared cell extracts (labeled on the left) and probed with anti-Rad50, anti-Mre11 or anti-Xrs2 antibodies (labeled on the right) by western blot. **(E)** Assessment of the Mre11-Rad50 and Mre11-Xrs2 interaction by the yeast-2 hybrid assay. Rad50, Mre11 and Xrs2 were expressed as a fusion with the Gal4-DNA Binding Domain (*pGBD; TRP1*) or the Gal4-Activation Domain (*pGAD; LEU2*) as indicated. Reporter activation assessed by growth in absence of histidine (Do-TLH) and adenine and histidine (Do-TLAH) were assessed in *WT*, *mre11-MRI-6A* and *mre11-E38K* mutants. Empty plasmids were included as a negative control.

Our structure of the MR-dsDNA complex revealed details of the interaction interface between Rad50 head domain and Mre11 capping domain, with five residues from Rad50 and six from Mre11 contributing to the interface (two alternate views in boxed panels of Fig. 2A). Rad50-E1155 makes hydrogen bond contacts with Mre11-K410 and Mre11-R412, while Rad50-D1167 interacts with Mre11-K322 and Mre11-R390. In addition, the interface is further stabilized by side chain hydrogen bond contacts between Rad50-E1243 and Mre11-R389. Rad50 residues E1155, D1167 and E1243 and Mre11 residues N387, R390, K410 and R412 are widely conserved (Fig. 2B, top and bottom, respectively), supporting their functional significance. Accordingly, mutation of these three Rad50 acidic residues (E1155, D1167 and E1243) to alanine (to create *rad50-MRI-3A*) imparts mild temperature dependent sensitivity to camptothecin (CPT) (Fig. 2C, top panel). Note that in the *rad50-MRI-3A* mutant, the Rad50-G1163 and -T1164 backbone interactions with Mre11-N387 and -R390 remain intact (boxed panels, Fig. 2A). All three possible double mutants from the *rad50-MRI-3A* triad exhibit modest CPT sensitivity at 37°, whereas only mild sensitivity was observed for single mutants at 37° (Fig. 2C, top panel).

*mre11-MRI-6A* contains alanine mutations of the six Mre11 residues noted above (K322A, N387A, R389A, R390A, K410A, and R412A). As Mre11-K410 and Mre11-R412 both form contacts with Rad50-E1155 (boxed panels, Fig. 2A), mutations of those Mre11 residues are *mre11-MRI-5A1* (*K322A N387A R389A R390A R412A*) and *mre11-MRI-5A2* (*K322A N387A R389A R390A K410A*). *mre11-MRI-6A* phenocopied *rad50-MRI-3A*, with markedly enhanced CPT sensitivity at 37°C (Fig. 2C, bottom panel). That *mre11-MRI-5A1* and − *5A2* exhibited equivalent sensitivity is consistent with the interpretation that Mre11-K410 and Mre11-R412 both contact Rad50-E1155 (boxed panels, Fig. 2A).

The CPT sensitivity of *rad50-MRI-3A*, *mre11-MRI-5A1* and *mre11-MRI-6A* mutants (Fig. 2C, lower panel) results from disruption of interaction with Xrs2 in those mutants. Co-immunoprecipitation from cells grown at 30°C or 37°C revealed that the abundance of Rad50-MRI-3A was strongly reduced both at 30°C and 37°C, but the interaction with Mre11 was intact at both incubation temperatures (Fig. 2D).

Similarly, Mre11-MRI-5A1, −5A2 and Mre11-MRI-6A proteins had only a modest decrement in Rad50 interaction as inferred from co-immunoprecipitations (Fig. 2D). To ask whether disruption of Mre11 dimerization accounted for the lack of Xrs2 interaction, a yeast two hybrid assay was carried out to determine if homodimerization was impaired. The data showed that Mre11-MRI-6A homodimerization was strongly compromised, as was interaction with Xrs2 and Rad50 (Fig. 2E).

### Protein-protein contacts involving Mre11 HLH domain with Rad50 head-proximal coiled-coil domain

**Figure 3:**
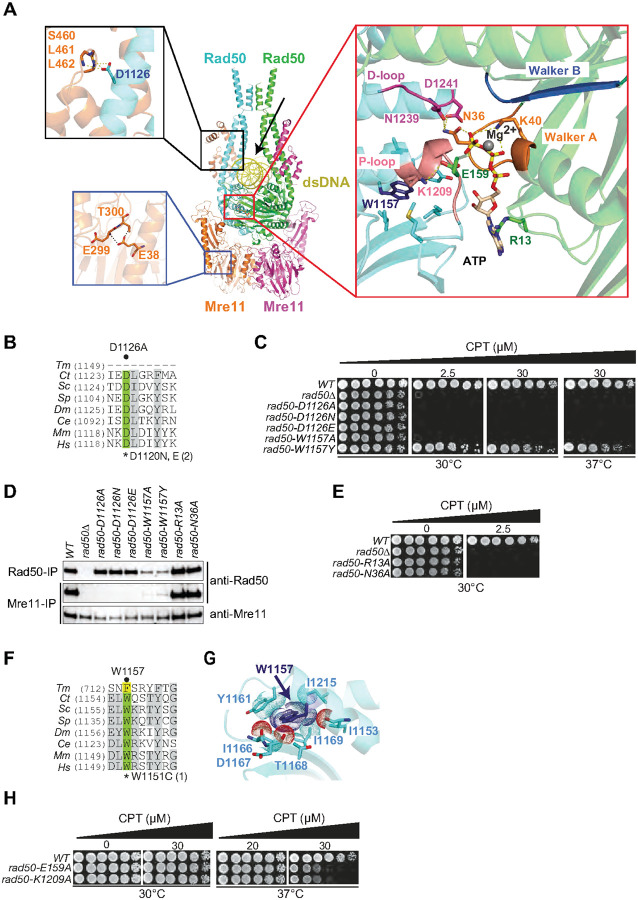
Critical residues in Rad50 coiled coils and Rad50 ATPase domain mediating Mre11 and Rad50-Rad50 interactions. **(A)** The boxed panels show protein-protein and ATP-protein contacts in the MR-dsDNA complex. (Top left panel) The Rad50 head proximal coil residue D1126 forms hydrogen bonds with the main chain of Mre11 HLH domain residues S460, L461 and L462. (Bottom left panel) Mre11 residue E38 forms hydrogen bonds with the main chain of Mre11 residues E299 and T300. (Right panel) Ligand-protein contacts between bound ATP and Walker A and B motifs of Rad50 in the complex. Encapsulation of Mg^2+^-coordinated ATP by conserved Walker A (orange) and Walker B (blue) motifs from one Rad50 D-loop (magenta) and P-loop (pink) from the other Rad50. Hydrogen bonds amongst ATP-protein and within the Rad50 dimer are shown as dashed lines. Critical residues mediating the Rad50-Rad50 interaction within this nucleotide binding domain (NBD) are shown. **(B)** Rad50-D1126 in the Rad50 head proximal coils is highly conserved. **(C)** CPT-sensitivities of *rad50*-mutants in D1126 and W1157 residues. **(D)** Mre11-Rad50 complex integrity in Rad50 coils (D1126A/N/E) and Rad50 ATPase (W1157A/Y, R13A, N36A) mutants assessed by co-immunoprecipitation. **(E)** Rad50 ATP-interacting residues R13 and N36 are critical for CPT-survival. **(F)** W1157 lining the ATP-binding pocket is highly conserved. **(G)** Positioning and Van der Waals contacts of W1157 within the hydrophobic pocket. **(H)** Minor CPT-sensitivities at 37°C of mutants in Rad50 residues (E159, K1209) mediating nucleotide-independent Rad50-Rad50 interaction.

Protein-protein contacts involving Mre11 HLH domain with Rad50 head-proximal coiled-coil domain Previous studies have noted that the Mre11 HLH domain lies across the Rad50 coiled coils proximal to the globular domain largely through hydrophobic interactions comprising approximately 970 Å^2 13,16^. Within the HLH domain, the structure revealed an interaction between the D1126 side chain and the backbones of Mre11-S460, -L461, and -L462 (Fig. 3A, top left boxed panel). Changing D1126, which is highly conserved (Fig. 3B), to alanine, asparagine or glutamate phenocopied the CPT sensitivity of the *rad50Δ* (Fig. 3C) and completely disrupted the Mre11-Rad50 association (Fig. 3D). These data indicate that the interface of the Rad50 coils, particularly the interactions of Rad50-D1126 with Mre11 HLH motif (Fig. 3A, top left boxed panel) are necessary and sufficient to retain the Rad50-Mre11 interaction.

Collectively, these data reveal the critical role of the Mre11 HLH domain, particularly that of Rad50-D1126 of the HLH domain (Fig. 3A, top left boxed panel), in promoting Mre11-Rad50 interaction. Single mutations distal to the Mre11 HLH domain, such as those involving the capping domain, imparted at best partial temperature sensitivity (Fig. 2C), indicating that those residues do not strongly influence the interaction of Mre11 and Rad50.

### Protein-protein and ATP-protein contacts centered on the ATP-binding pocket of Rad50

In addition to homodimerization at the apical Zinc hook domain, Rad50 homodimerization is also mediated by Walker A and B motifs, with the Walker A from one protomer interacting with Walker B from the other^17^. This interaction is stabilized by ATP coordination between each Walker A and B pair such that two ATP molecules reside within the interface.

Within the Walker A motif, Rad50-R13 forms a hydrogen bond with the adenosine moiety of ATP and Rad50-K40 coordinates with the b and g phosphates of ATP, whereas Rad50-N36 interacts with both the ATP g-phosphate and Rad50-D1241 in the D loop of the opposing Walker B motif (Fig. 3A, right boxed panel). Mutation of the corresponding residues in bacteriophage T4 Rad50 (gp46) severely reduces ATP binding affinity and hydrolysis, as well as Mre11 (gp47) dependent nuclease activity^17^. Consistent with those data, *rad50-R13A* and *rad50-N36A* phenocopy the CPT sensitivity of *rad50Δ* (Fig. 3E). Notably, this outcome was not due to disruption of the Mre11-Rad50 interaction, as co-immunoprecipitation of these Rad50 mutants and Mre11 was indistinguishable from WT (Fig. 3D).

Rad50-W1157 is conserved from *S. cerevisiae* to humans (Fig. 3F). It lies just adjacent to the ATP binding pocket in the Walker B motif (Fig. 3A, right boxed panel). The W1157 residue extends into a hydrophobic pocket within the same protomer where it makes multiple van der Waals contacts (Fig. 3G). It appears critical that this residue is aromatic (Fig. 3F). It is substituted with phenylalanine in *T. maritima* (Fig. 3F), and whereas *rad50-W1157A* is as CPT sensitive as *rad50Δ*, *rad50-W1157Y* is only minimally sensitive to CPT (Fig. 3C). The CPT sensitivities observed are associated with destabilization of Rad50 itself, as well as its interaction with Mre11. Co-immunoprecipitation experiments reveal that the abundance of Rad50-W1157A and -W1157Y is reduced, but some residual interaction with Mre11 is observed only for Rad50-W1157Y (Fig. 3D). Similarly, the interaction between Rad50-E159 and -K1209 (Fig. 3A, right boxed panel) plays a less significant role in Mre11 complex function, as *rad50-E159A* and *rad50-K1209A* exhibit mild CPT sensitivity only at 37° (Fig. 3H).

Mre11 and Rad50 are each stabilized by intramolecular interactions within individual domains. Mre11-E38 forms side chain and backbone contacts with Mre11-T300 and E-299 within the same protomer (Fig. 3A, lower left boxed panel). In a previous study, the *mre11-E38K* mutation was modeled in yeast after the corresponding human residue was found to be recurrently mutated in ovarian and endometrial cancers. *mre11-E38K* exhibited strongly reduced levels of Mre11-E38K protein and sensitivity to CPT^18^. As above, a two hybrid assay showed that Mre11-E38K homodimerization is compromised, as is interaction with Rad50 and Xrs2 (Fig. 2E). This indicates that disruption of the contacts with Mre11-T300 and -E299 alters the overall structure of Mre11 and thereby has global effects on the structure of the Mre11 complex.

### Protein-dsDNA contacts

**Figure 4:**
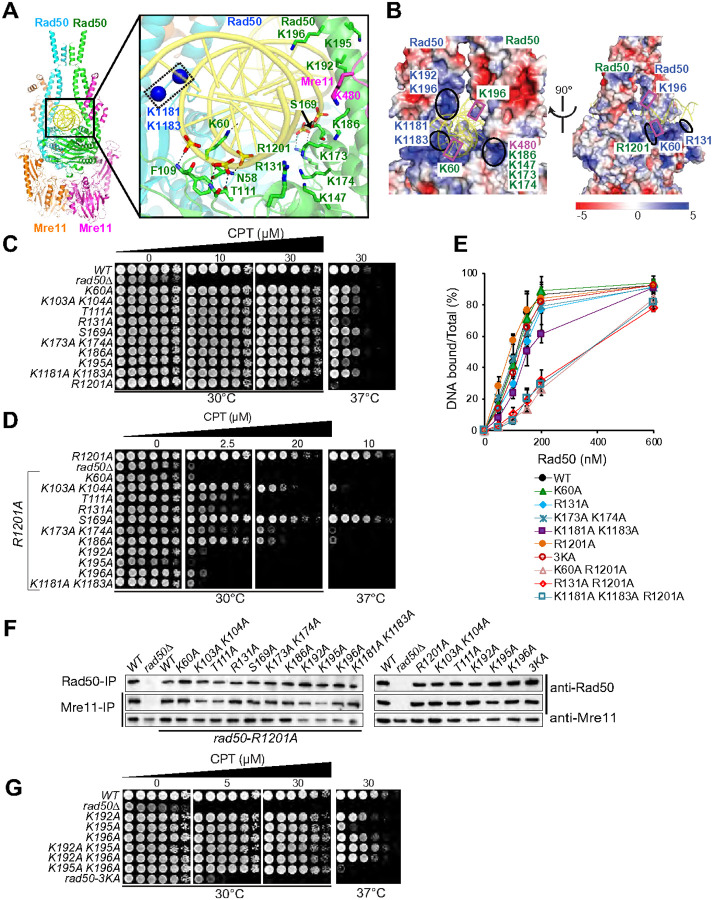
Mutational assessment of Rad50 residues involved in interactions with the dsDNA phosphodiester backbone. **(A)** The boxed panel shows details of intermolecular protein-DNA contacts (dashed black lines) between the side and main chain residues of Rad50 and phosphates of dsDNA in the complex. The basic side chains lining the positively-charged DNA binding pocked are shown in stick representation, except for the untraceable side chains of Lys1181 and Lys1183, which are shown in ball representation. **(B)** The dsDNA is encapsulated within a basic channel lined primarily by basic lysine and arginine residues. Two views rotated by 90° of the surface electrostatic potential of MR in the region surrounding the bound dsDNA in the complex, with positively charged basic surfaces labeled and shown in blue and green from each Rad50 protomer. **(C)** CPT-survival of *rad50* mutants targeting potential dsDNA binding residues assessed at 30°C and 37°C. **(D)** CPT-survival of *rad50* mutants targeting potential dsDNA binding residues (panel C) in *rad50-R1201A* background assessed at 30°C and 37°C. **(E)** Quantitation of DNA binding of WT and Rad50-mutant proteins. Indicated amounts (0–600 nM) of purified Rad50 proteins (see Figure S4A) were incubated with 5 nM 83-mer dsDNA (same substrate as used in cryo-EM) in the presence of 2 mM cold ATP, 0.1 mM y^32P^-ATP and 5 mM MgCl_2_. Bound- and free-substrates were separated by the electrophoretic mobility assay (EMSA). Examples of EMSA gels are given in Figure S4B. Error bars denote standard deviation from at least three experiments. **(F)** Mre11 complex integrity of Rad50-dsDNA binding residue mutants assessed by Rad50-and Mre11-co-immunoprecipitation (labeled Rad50-IP; Mre11-IP) and Rad50 and Mre11 Western blotting (anti-Rad50; anti-Mre11). The integrity of *rad50*-single and double mutants were assessed both in *rad50-R1201A* (blot on left side) and *WT(R1201)*-background (blot on right side). **(G)** CPT-survival of *rad50-K192A, K195A* and *K196A* single, as well as double mutants and triple mutant *(rad50–3KA)* assessed at 30°C and 37°C.

The DNA binding contacts of the eukaryotic Rad50-dsDNA complex from *C. thermophilum* have been previously described^19^. In our structure of the *S. cerevisiae* MR-dsDNA complex, we noted that 18-bp of dsDNA [modeled as an (dA)_n_-(dT)_n_ duplex] is encapsulated by elements of the pair of Rad50 domains in the MR-dsDNA complex (Figs. 1B, 1C and 4A). The 18-bp DNA footprint of MR is consistent with that seen for the *C. thermophilum* Rad50-dsDNA^19^ and *E. coli* MR (SbcCD)-dsDNA^10^ complexes. The dsDNA is positioned above the pair of Rad50 head domains and sandwiched between the pair of Rad50 head-proximal coiled coil arms, anchored in place through a network of hydrogen bonding interactions. We can trace intermolecular hydrogen bonds between Rad50 side chains (-T111, -S169 and -R1201) and main chain (-N58, -F109, -T111 and -S169) with backbone phosphates of bound dsDNA (Fig. 4A, boxed panel). Notably, the side chain of Rad50-K60 is inserted deep into the minor groove of bound dsDNA and is likely positioned to form hydrogen bonds with bases of dsDNA. The bound dsDNA is positioned within a basic channel lined primarily by lysine side chains originating from the pair of encapsulating Rad50 domains in the complex (Fig. 4B).

It is well established that defects in dsDNA binding by Rad50 confer sensitivity to clastogenic insult. Therefore, we assessed CPT sensitivity of alanine mutants of dsDNA-interacting residues described above. As with alterations of protein-protein interfaces, phenotypic assessment was carried out at 30°C and 37°C, because partial destabilization of Rad50 dsDNA binding may confer a temperature-sensitive phenotype.

Basic residues within the Rad50 channel that point towards the dsDNA phosphate backbone and are situated within 2 to 4 Å were changed to alanine (Fig. 4A, boxed panel). With the exception of *rad50-R1201*, single mutations of these residues had virtually no impact on CPT sensitivity (Fig. 4C). We purified Rad50 WT and mutant proteins from yeast cells (Figure S4A) and assessed Rad50 ATP-dependent dsDNA binding by EMSA using a radiolabeled 83-mer dsDNA substrate (Figure S4B). Rad50-K60A, -R131A, -K173A K174A, -K1181A K1183A and -R1201A exhibited essentially WT dsDNA binding activity (Figs. 4E and S4B), consistent with the lack of CPT sensitivity (Fig. 4C). Some of these residues are conserved from archaea to human and were previously shown to interact with dsDNA^13,15,19^. They include Rad50-K60, -K103, -K104, -R131 and -R1201 and were previously assessed phenotypically following charge reversal substitution with glutamate and shown to impart strong defects in DNA repair^15,19^. The mutants shown in Fig. 4C were next combined with *rad50-R1201A*. All of the mutant combinations were nearly as CPT sensitive as *rad50Δ*, with the exception of *rad50-K103A K104A R1201A*, *rad50-K186A R1201A* and *rad50-S169A R1201A* (Fig. 4D). As expected, this was correlated with reduced dsDNA binding (Figs. 4E and S4B).

Decrements in DNA binding were not due to loss of Mre11 complex integrity (Fig. 4F). Rad50 protein levels were comparable in any single and R1201A double mutants. Interactions between Rad50 and Mre11 were slightly reduced in *rad50-K103A K104A R1201A*, *rad50-T111A R1201A* and *rad50-K192A R1201A* and were partially impacted in *rad50-K195A R1201A* (Fig. 4F). Nevertheless, these data support the view that dsDNA binding defects underlie the phenotypes observed.

### A transient Rad50 DNA binding site?

As described below, Molecular Dynamics (MD) simulations suggested that residues of Rad50 somewhat distal to the dsDNA binding domain (Rad50-K192, -K195 and -K196) in the 3.2 Å structure of the MR-dsDNA complex (Fig. 4A, boxed panel) may transiently engage DNA prior to the binding mode observed in the structure.

The Rad50-K196 side chain points towards the dsDNA at a distance of 4.3 Å from the backbone phosphate, while Rad50-K192 and -K195 positioned on the same helix, are rotated away from the DNA phosphate backbone by a distance of 5.3 Å and 10.1 Å, respectively (Fig. 4A, boxed panel). Rotation of the coiled coils, suggested by MD simulations (see below), may bring Rad50-K192 and -K196 into contact with the dsDNA, at least transiently, while K195 has a structural role in positioning K192 and K195 for DNA-interaction.

To address the significance of these residues, alanine substitutions were carried out and assessed phenotypically. The single alanine substitutions had very little impact on CPT sensitivity. In contrast, the triple mutant *rad50-K192A K195A K196A* (hereafter referred as *rad50–3KA*) was almost as sensitive as *rad50Δ* (Fig. 4G). This strong CPT-sensitive phenotype was not due to a compromised Mre11 interaction, nor disruption of Rad50–3KA dsDNA binding, as WT-levels of Rad50–3KA were immunoprecipitated with Mre11 (Fig. 4F), and Rad50–3KA dsDNA binding was unaffected *in vitro* (Figs. 4E and S4B).

### Intragenic suppressors of rad50–3KA

**Figure 5:**
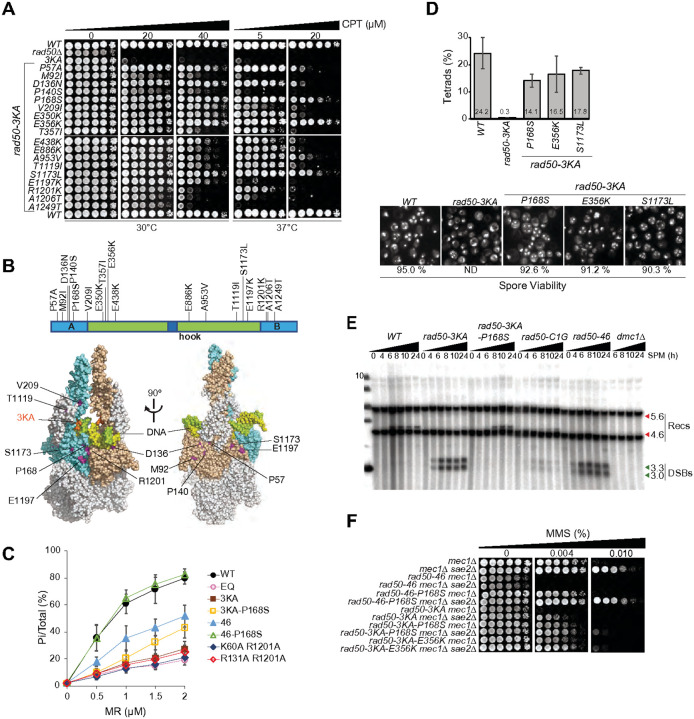
Intragenic suppressors of *rad50–3KA* CPT-survival alleviate the Rad50–3KA ATPase defect. **(A)** CPT-survival of 18 intragenic *rad50–3KA* isolated in a genetic screen. Plasmids of the original screen suppressor clones were recovered, sequenced and re-transformed in a *rad50D* strain. CPT survival after retransformation is shown. Plates were either incubated at 30°C or 37°C. **(B)** Localization of the *rad50–3KA* suppressor mutations in Rad50 primary sequence (top) and on the Rad50 structure (bottom). Note that the P168S suppressor previously isolated in a screen suppressing *rad50–46* hook mutant phenotypes^20^ was also independently isolated for *rad50–3KA* here. **(C)** Rad50-ATPase activity of purified WT and mutant MR complexes. Indicated concentrations (0–2 μM) of MR-proteins were incubated in the presence of both ss and dsDNA in a buffer containing cold and hot ATP and MgCl_2_. The hydrolyzed radiolabeled phosphate was separated from g^32^-ATP by thin-layer chromatography. Error bars denote standard deviation from at least three experiments. **(D)** Meiosis in W303+ background of *WT, rad50–3KA* without and with suppressors (*P168S, E356K* and *S1173L*). Sporulation efficiencies (top panel; percentage of Tetrads in sporulated cultures) and spore viability (bottom panel; determined by tetrad dissection) are shown. Representative pictures of DAPI-stained sporulated cultures are shown. The spore viability of *rad50–3KA* without suppressors could not be determined (ND) due to the lack of tetrads. **(E)** Meiotic DSB formation and repair by meiotic recombination at the *HIS4-LEU2 hotspot* by southern blot. The migration level of the crossover recombinant fragments (Recs, recombinants; 5.6 kb and 4.6 kb, red arrows) above and below the parental band^24, 35^ and the unprocessed 3.3 kb and 3.0 kb meiotic double-strand break fragments (DSB, green arrows) are denoted. Cells were cultivated in sporulation media (SPM) for 0, 4, 8, 10 and 24 (*WT, rad50–3KA, rad50–3KA P168S, rad50-C1G* and *rad50–46*) or for 6, 8, 12 and 24 hours (*dmc1Δ*). **(F)** Cell survival of RAD50 WT, *rad50–46, rad50–3KA without and with P168S or E356K suppressor mutants* in a Mec1-and/or Sae2-deficient background. All strains were in the *mec1Δ sml1Δ background*.

We performed an intragenic suppressor screen to identify mutations that could bypass the *rad50–3KA* phenotype, perhaps by promoting the handoff of dsDNA from Rad50 to Mre11, an event that would not be detectable *in vitro*. A centromeric plasmid containing the *rad50–3KA* ORF was chemically mutagenized and transformed into *rad50Δ/RAD50* diploid cells. Following sporulation, random *rad50Δ* spores containing the mutagenized plasmid were recovered. Among 960 colonies analyzed for growth on selective plates containing 30 μM CPT, 18 clones showed increased resistance to CPT relative to *rad50–3KA* (Fig. 5A).

Ten of the eighteen suppressors were located either within the Walker A and B motifs or in the proximal coiled coils (Fig. 5B, top panel); Walker A (P57A, M92I, D136N, P140S, P168S), Walker B (R1201K, A1206T, A1249T), the N terminal coils (V209I) and the C terminal coils (T1119I). Two are located on b hairpin proximal to head domains (S1173L, E1197K), while the remaining six lie within the coils distal to the Walker A and B motifs (E350K, E356K, T357I, E438K, E886K, and A953V). With the exception of P168S and R1201K, most suppressors in Walker A/B motifs were surface exposed and localized > 10 to 20 Å below the bound dsDNA (Fig. 5B, bottom panel), indicating that suppression of *rad50–3KA* can be effected at mid- and long-range distances.

Mutations in the coiled coil regions as well in the Walker A and B motifs were previously shown to suppress the phenotype of a mutation in the Rad50 hook domain, *rad50–46*. Notably, P168S, which was recovered in the screen for *rad50–3KA* suppressors was also previously recovered in an independent screen for *rad50–46* intragenic suppressors^20^.

### Defects in Rad50 ATPase activity

ATP hydrolysis mediates the transition from the closed to the open form of the Mre11 complex^13^, which is a prerequisite for the engagement of dsDNA in the active site of Mre11^6^. The *rad50–3KA* pronounced CPT sensitivity (Fig. 4G) and normal dsDNA binding (Fig. 4E), could therefore be attributable to a defect in ATP hydrolysis. To test that interpretation, we purified WT and mutant MR complexes (Figure S4C) and measured ATP hydrolysis as a function of MR concentration (0–2 μM) after 90 minutes of incubation with ATP- g^32^P (Figs. 5C and S4D). We observed at least a 50% reduction in ATPase activity of Rad50–46 compared to WT, whereas Rad50–3KA, Rad50-K60A R1201A and Rad50-R131A R1201A were as defective as the Rad50-EQ mutant (Fig. 5C).

P168S reverted the CPT sensitivity of both *rad50–46* and *rad50–3KA* to that of *WT* (Figure S5A). Although the ATPase activity specified by Rad50–46 is 2.5 higher than that of Rad50–3KA (Fig. 5C), P168S suppressed each by the same magnitude – roughly two fold. This result indicates that Rad50–46 and Rad50–3KA are both defective in the same DNA repair mechanism dependent on Mre11 nuclease activity and Sae2 (Figures S5B and S5C).

In meiosis, the Mre11 nuclease activity is required to remove Spo11 from meiotic DSB ends^21,22^. *rad50–46* accumulates unprocessed meiotic DSBs due to failure to remove Spo11. P168S rescued *rad50–46* meiotic phenotypes essentially to *WT* levels^20^. Meiotic progression in *rad50–3KA* was assessed (Fig. 5D, top panel). Essentially no tetrads could be detected in *rad50–3KA* sporulated cultures, and DAPI signals appeared heavily fragmented as previously observed in *spo11Δ/Δ* cells and resulting in meiotic chromosome missegregations^23^. This meiotic defect was rescued almost to *WT* levels by all three *rad50–3KA* suppressors tested (P168S, E356K and S1173L; Fig. 5D, top panel), with both sporulation efficiencies (14–17%) and spore viabilities (90–93%), comparable to *WT* (24% and 95% respectively; Fig. 5D, bottom panel).

We asked whether *rad50–3KA* meiotic defect is due to a failure to remove Spo11 from meiotic DSBs. A *rad50–3KA* strain without and with *P168S* was constructed and used to visualize unprocessed meiotic DSBs and meiotic DSB repair products (recombinants)^24^. P168S rescued the *rad50–3KA* meiotic DSB processing defect as previously observed for *rad50–46*^20^, evident by the bands now appearing to represent the meiotic recombinants products (Fig. 5E). A *rad50* hook mutant defective in zinc coordination (*rad50-C1G*), and a Rad50 ATPase catalytic mutant (*rad50-E1235Q*), failed to efficiently form meiotic DSBs and did not exhibit appreciable unprocessed DSBs or recombinants (Fig. 5E and data not shown), and did not produce viable spores (data not shown).

The Rad50–3KA ATPase defect is more severe than that of Rad50–46 (Fig. 5C), making it likely that additional functions may be compromised. The *rad50–46* mutant is defective in Tel1 activation^20^, as inferred from the inability of *rad50–46 sae2Δ* to suppress Mec1 deficiency^25^. That phenotype is suppressed by P168S (Fig. 5F). Defective Tel1 activation results in telomere shortening, and comparable telomere lengths were observed in *rad50–46* and *rad50–3KA*. Whereas P168S restored normal telomere length to *rad50–46*, it failed to do so in *rad50–3KA* (Figure S5D). P168S suppresses the ATP hydrolysis defects in *rad50–46* and *rad50–3KA* by approximately the same magnitude (two fold), but the latter double mutant remains two fold less active than the former. These data indicate that a threshold of ATPase activity is required for Tel1 activation (Fig. 5F).

### Molecular Modeling and MD Simulations of MR bound to dsDNA

**Figure 6:**
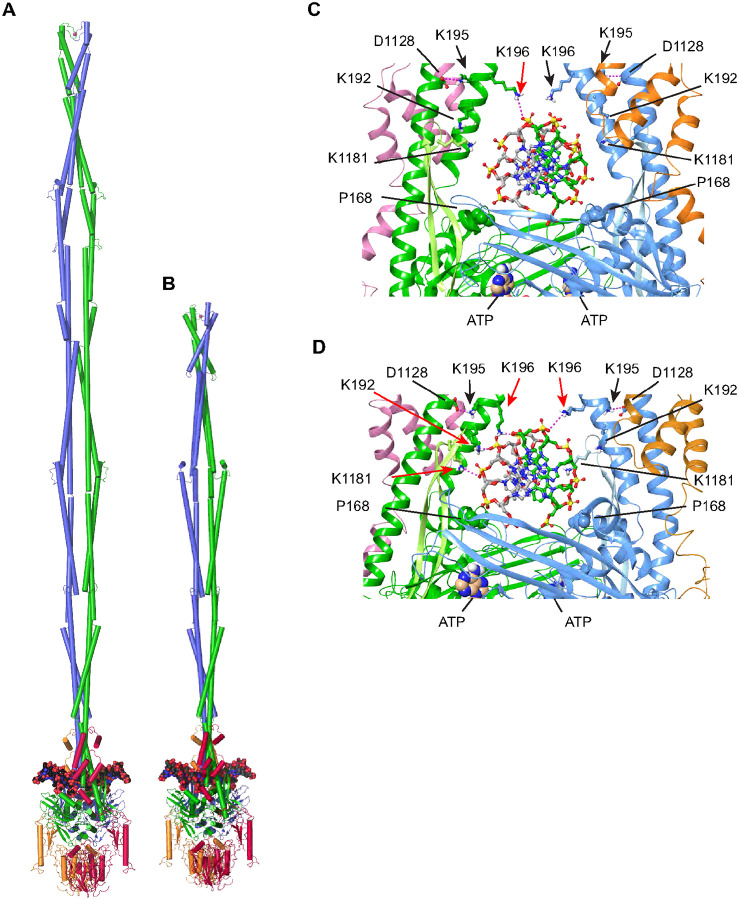
Full-length and truncated coiled-coil version of the dsDNA bound *S. cerevisiae* MR complex and details of MR-DNA interactions during MD simulation. **(A, B)** The topology of the full-length (panel A) and coiled-coil truncated (panel B) DNA bound MR complex. **(C, D)** Details of amino acid K192, K196, K1181, D1128, and P168 side chain conformations before (panel C) and after (panel D) 30 nsec of MD simulations. Salt bridges are shown as dashed lines. The side chain of P168 is shown in space-filling representation. Lysine residues involved in hydrogen bond formation are shown by red arrows.

Only a small segment of the head-proximal coiled coils of Rad50 were resolved in the cryo-EM structure of the *S. cerevisiae* MR-dsDNA complex (Figs. 1B and 1C). Thus, we constructed a model of full-length *S. cerevisiae* MR-dsDNA complex (Fig. 6A), using the computational protocol described in the Methods section (Figures S6A-S6L and S7A-S7C).

To facilitate MD simulations, we constructed a model of the MR-dsDNA complex containing truncated Rad50 coiled-coils ending with the Zn hook (Fig. 6B), using the protocol described in the Methods section (Figures S7D and S7E).

The MR-dsDNA complex containing truncated coiled-coils was subjected to MD simulations (Figs. 6C and 6D). We observe a coaxial rotational movement of the Rad50 coiled coils along their axes in the WT MR-dsDNA complex during the 30 ns simulation (Movie 1). The dsDNA is initially positioned with K196 of individual Rad50 monomers hydrogen bonded to the phosphates of opposing strands of the DNA duplex (Fig. 6C). At the end of 30 ns, K192 and K196 (on an a-helix) and K1181 (on a b-hairpin) from one Rad50 monomer (in green) form an asymmetric interaction with consecutive phosphate backbone of one strand of the DNA duplex (Fig. 6D). The mild CPT sensitivity of K195A (Fig. 4G) is likely due to the loss of the salt bridge between the side chains of N-terminal K195 and C-terminal D1128 of Rad50 (Figs. 6C and 6D) that provides rigid support for the interactions of K192 and K196 with consecutive phosphates on one DNA strand (Fig. 6D). It should also be noted that Rad50 b-hairpins (Thr1168 to Lys1193; shown in lighter color in Figs. 6C and 6D) move towards the same minor groove of dsDNA from two opposite Rad50 monomers, albeit closer to a different DNA strand, at the end of the 30 ns MD simulation, as a result of which K1181 (on the green colored b-hairpin) is positioned to form a hydrogen bond with the backbone phosphate (Fig. 6D).

### ATP-hydrolysis is restored in Rad50–3KA P168S.

**Figure 7:**
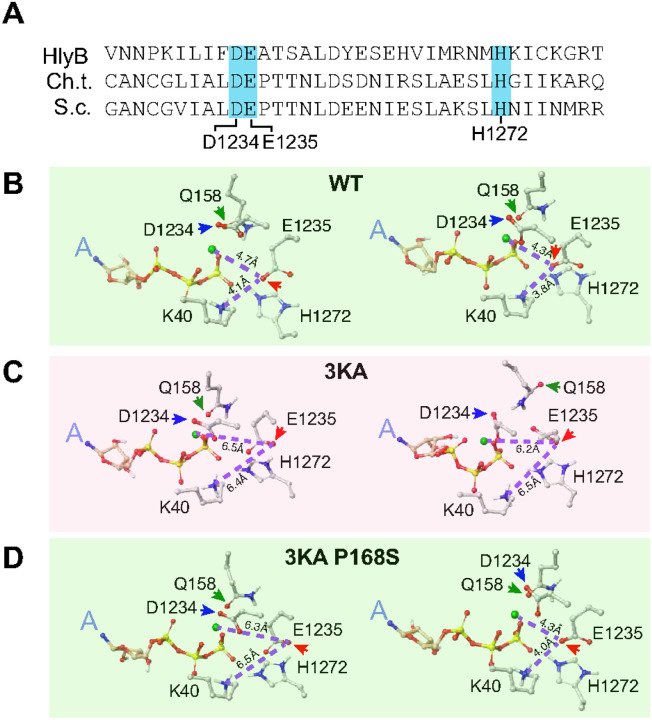
Geometric characterization of residues promoting ATP hydrolysis **(A)** Sequence alignment of the catalytic residues of three ABC ATPases. The conserved D, E and H residues in ABC-transporters of Hemolysin B, *C. thermophilum* and *S. cerevisiae* are highlighted in blue background. **(B-D)** The conformations of catalytically-competent amino acid side chains at the beginning (left panel) and end (right panel) of 30 nsec MD simulation of the dsDNA-bound truncated MR complex: WT (panel B), Rad50–3KA (panel C), and Rad50–3KA P168S (panel D). Dashed lines indicate the distances between negatively charged oxygen (as computed by the SCRODINGER program) on the catalytically competent E1235 and the positively charged Mg^2+^ and ammonium group of K40. The negatively charged carboxylate oxygens of D1234 and E1235 residues are shown by blue and red arrows, respectively. The partially negatively charged amide oxygen of Q158 is shown by a green arrow.

MD simulations were carried out using coiled coil truncated WT, Rad50–3KA and Rad50–3KA P168S MR-dsDNA complexes (Fig. 7). Hydrolysis of the ATP b-g phosphodiester bond occurs via nucleophilic attack of an activated hydroxyl ion of a water molecule, resulting in hydrolysis of the b-g bond and release of the g-phosphate^26,27^. Conserved Rad50-D1234, -E1235, -H1272 and Q158 line the ATP-binding pocket and mediate hydrolysis (Fig. 7A).

We carried out 30 ns MD simulations on truncated versions of WT (Fig. 7B), Rad50–3KA (Fig. 7C) and Rad50–3KA P168S (Fig. 7D) MR-dsDNA complexes. Side chain alignments of Rad50-E1235 (red arrow), -D1234 (blue arrow) and -Q158 (green arrow) with triphosphate chain of bound ATP are shown before and after 30 ns. Similar side chain alignments of D1234, E1235 and Q158 lining the ATP pocket were observed for WT (Fig. 7B, right panel) and Rad50–3KA P168S (Fig. 7D, right panel) at the end of the 30 ns. The same side chains align differently in the Rad50–3KA MR-dsDNA complex at the end of the simulation (Fig. 7C, right panel). The relevant distances between the side chains and Mg^2+^ before and after the 30 ns MD simulation and the sum of the differences in their conformational changes are summarized in Table S2. Notably, the partially negatively charged amide oxygen on the side chain of Q158 retains its position in WT (Fig. 7B, right panel) and Rad50–3KA-P168S (Fig. 7D, right panel) but undergoes a large shift away from the Mg^2+^ at the end of the 30 ns simulation in Rad50–3KA (Fig. 7C, right panel). In contrast, the negatively charged atoms in the Rad50–3KA P168S have become closer to the catalytic center by 11.1 Å (Table S2). Hence, the ATP hydrolysis mechanism is effectively restored to that of wild type Rad50 in Rad50–3KA P168S.

### Rotation of Rad50 coiled coils

The 30 ns MD simulations of WT (Movie 1) and Rad50–3KA P168S (Movie 2) also revealed a clockwise coaxial rotation of the coiled coils (as viewed from the Rad50 globular domain toward the Zn hook). Clockwise rotation was also observed for Rad50–3KA, but the trajectory deviated from a coaxial rotation (Movie 3) revealing the impact of Rad50–3KA on the coiled coils. Notably, the same direction of rotation was also observed in the WT MR-dsDNA complex with full-length Rad50 coiled coils; thus, it was not the result of the truncation. Morphing the transition from DNA-free *C. thermophilum* MR complex with rod-like coiled coil^11^ to dsDNA-bound complex presented here is also accompanied by clockwise rotation of the coiled coils (data not shown).

## DISCUSSION

Here we report on a cryo-EM structure of the *S. cerevisiae* MR-dsDNA complex at 3.2 Å resolution, partially augmented by molecular modeling and molecular dynamics simulations. Our detailed structure-function analysis of protein mutants revealed new features of the Rad50-dsDNA interaction interface, as well as novel inter- and intra- molecular protein-protein contacts that are critical for the assembly and dynamics of the MR-dsDNA complex. Data obtained over the last ten years indicate formation of two major states of the MR-dsDNA complex, namely an ATP bound “closed” complex with dsDNA bound by Rad50 dimers important for DNA end stabilization/sensing and activation of the Tel1 checkpoint kinase, and an “open” complex which forms upon ATP hydrolysis, and in which the Mre11 nuclease active site is accessible to the dsDNA substrate^28^.

The structure of the stable *S. cerevisiae* MR-dsDNA complex presented here appears to represent a “closed” complex, having a rod-shaped structure that is similar to that recently reported for the *C. thermophilum* MRN complex in presence of ATPgS, but without DNA^11^. Of the 83-mer dsDNA used for complex formation in our study, 18 base pairs of duplex DNA are bound in a central basic DNA binding groove formed by the ATP-bound Rad50 dimer, with the Rad50 coils encapsulating the dsDNA from the top (Figs. 1B, 1C, 4A and 4B). The dsDNA length and Rad50 DNA interacting residues align well with the previously reported crystal-structure of the dsDNA bound *T. maritima* Rad50^19^, with the exception that the coils remained open in that structure. It is thus conceivable that the closed structure presented herein and that of the *T. maritima* represent different stages in the engagement of dsDNA by Rad50.

The distal coiled coils of Rad50 were not visible in our cryo-EM structure of the MR-dsDNA complex (Figs. 1B and 1C), but molecular modeling (Fig. 6A) suggests that the full-length coiled coils are in a closed configuration, essentially spanning the segment from the globular to the hook domain but punctuated with short flexible regions which are analogous to the “hinge” loop (CGSQD) identified in the human Rad50 hook structure^12^. This is consistent with atomic force microscopy (AFM) studies of the human MR complex which showed that upon dsDNA binding, the coiled coils adopt a rod-shaped parallel conformation^29^. Hence, it appears that at least partially opened coils are required for initial DNA binding by Rad50, likely loaded proximal to the head domains of Rad50, in agreement with our previous Rad50 hook structure, as well as AFM data regarding the human complex^12,30^.

The major point emerging from this study is that the coiled coils influence Rad50 ATP hydrolysis. It is clear from previous work that ATP hydrolysis by Rad50 governs the transition from the “closed” to “open” forms of the complex^13,28^. Hence, there is a reciprocal relationship between ATP hydrolysis and the disposition of the coiled coils. The Rad50 ATPase in Rad50–3KA is essentially equivalent to the Rad50-E1235Q hydrolysis defective mutant (Fig. 5C), and blocks cleavage of Spo11 from meiotic DSB ends (Fig. 5E). Spo11 cleavage, and therefore the ATPase activity of Rad50–3KA appears to be restored by E365K (in the coiled coils) and S1173L (in the conserved b-hairpin loop^11^), both of which suppress the spore inviability of Rad50–3KA (Figs. 5C and 5D). Additional suppressors or Rad50–3KA were identified in the coiled coils underscoring the mid- and long- range influence of the coiled coil domain (Figure S8). In this regard, it is perhaps notable that recent proteomic data reveal multiple Tel1 phosphorylation sites exclusively within the Rad50 coiled coils^31^, suggesting a mechanism for transient modulation of coiled coil behavior.

A previously described Rad50 hook domain mutation, *rad50–46* exhibits CPT sensitivity that was suppressed by two mutations in the coiled coils, N607Y and N873I^20^. Given the role of Rad50 ATPase activity in potentiating Mre11 nuclease function, we presume that the ATPase defect in *rad50–46* (Fig. 5C) underlies both the CPT sensitivity and defects in meiotic DSB processing observed in that mutant^20^. In addition to suppressors in the coiled coils, both *rad50–3KA* and *rad50–46* are suppressed by P168S in the Rad50 head domain. It is remarkable that *rad50–46* and *rad50–3KA*, which are at least 300 Å apart, are both suppressed by P168S which lies within the Walker A domain and very far from the hook domain.

Extensive mutational analyses and MD simulations reveal previously unappreciated dynamics of the coiled coils as they engage DNA. The simulations suggest that the Rad50 coiled coils undergo a clockwise coaxial rotation (Movie 1) which coincides with transient contacts between Rad50 K192 K195 K196 (Rad50–3K residues) and dsDNA. This rotation appears to impel the DNA deeper into the cleft formed below the coiled coils, ultimately leading to the disposition captured in the cryo-EM structure of the complex. This “zippering” motion of the coiled coils around DNA is reminiscent of that recently described for the SbcCD complex^10,11^. The Rad50–3KA containing Mre11 complex does not exhibit defects in DNA binding *in vitro*, consistent with the view that the interaction of dsDNA with Rad50 K192 K195 K196 may be transient and does not represent a stable DNA binding surface. In the *C. thermophilum* MRN complex structure without dsDNA, residues corresponding to Rad50 K192 K195 K196 face into the channel between the coiled coils and make contacts with a, at primary sequence, poorly conserved b-hairpin loop^11, 19^, but present in all Rad50 structures so far. That most of the b-hairpin loop is no longer visible in the structure of the MR-dsDNA complex presented here, suggesting that it folds away upon DNA binding or becomes disordered (Movie S1).

The residues at the Rad50-dsDNA interface are highly conserved; many residues assessed here were previously reported residues in *E. coli* and *T. maritima*^9,10,19^, and are in close proximity to the 18 base pair DNA duplex in our structure. Previous studies made charge reversal mutants in various yeast Rad50 DNA binding residues, resulting in CPT sensitivity and in reduced DNA binding *in vitro*^15,19^. Alanine substitutions of these residues were individually inconsequential, as mutants showed WT CPT survival and dsDNA binding (Figs. 4C and 4E). In agreement with these previous reports, when two or more residues that included R1201A were mutated (Fig. 4D), these mutants were strongly sensitized to CPT almost to the level of *rad50Δ* and showed reduced dsDNA binding *in vitro* (Fig. 4E) underscoring the fact that multiple contacts between Rad50 and dsDNA contribute to DNA binding. DNA binding stimulates Rad50-ATPase. Accordingly, we found that both Rad50-K60A R1201A and Rad50-R131A R1201A showed ATPase activities reduced to the levels of Rad50-E1235Q ATPase deficient mutant (Fig. 5C).

As noted above, at the end of 30 ns MD simulations, three lysine residues of Rad50 were identified that either directly interacted with the phosphodiester backbone of the dsDNA (K192 and K196) or stabilized the coils through interaction between K195 and D1128 (Fig. 6D); however, in our structure, these are too far situated (> 4.5 Å) to reveal a DNA interaction. Both K192 and K195 are highly conserved in evolution and are found to be repeatedly mutated (24 tumors in MSK IMPACT analyses^32^), while K196 is not conserved in mammals. Within the *E. coli* MR complex, two lysine residues (K194 and K890) interact with DNA and charge reversal mutants showed also reduced DNA binding^10^. The crystal structure of *T. maritima* Rad50-dsDNA revealed three lysine residues within the coils (K175, K178 and K182) that interacted with the DNA and charge reversal mutations also resulted in reduced DNA binding^15^. Except K182, which aligns with K192, these residues do not align with the *S. cerevisiae* K192, K195 and K196, but are located in the same part of the coils (a7 helix). In this context, we propose that the *S. cerevisae* Rad50 K192, K195 and K196 residues, amongst others, allow the coils to properly position the duplex DNA in the basic DNA binding groove which is required to activate the Rad50 ATPase. The failure to activate the Rad50 ATPase would in turn block opening of the complex to provide DNA access to the Mre11 catalytic center^10,33^.

Collectively, the data reported here strongly illustrate the influence of the Rad50 coiled coil domains on the enzymatic activities specified within the globular domain of the Mre11 complex. Moreover, the extensive structure-based functional analyses presented provide a highly granular view of the interfaces and residues that mediate complex assembly as well as function.

The extended coiled coil structure is a conserved feature of Rad50 across all clades of life. This begs a question not addressed here: why are the coiled coils so long? We showed previously that shortening of the coiled coils compromised Mre11 complex function, even when Rad50 with shortened coils was co-expressed with Rad50 having a corresponding increase in coiled coil length^34^. This indicates that the coiled coil length *per se* is not paramount. Recent work suggests that bridging of sister chromatids during DSB repair is mediated via coiled coil interactions in *trans*^11^. That mode of bridging with *S. cerevisiae* Rad50 could span up to 120 nm. That distance is far greater than would be required to accommodate two 30 nm fibers of heterochromatic stretches of chromatids, but it is conceivable that loops or other structures of sister chromatids in euchromatic regions could necessitate a longer coiled coil structure to effect tethering during DSB repair. As the granularity of the globular domain structure increases, and the influence of the coiled coils becomes clearer, the need to understand the dynamics and structure(s) relevant to the Rad50 coiled coils becomes more acute.

## Data Availability

The atomic coordinates of the *S. cerevisiae* dsDNA-bound MR^EQ^ complex have been deposited in the Research Collaboratory for Structural Bioinformatics Protein Data Bank with the code 9BI4. Cryo-EM density maps have been deposited in the Electron Microscopy Data bank with accession code EMD-44558. The atomic coordinates of an Apo MR^EQ^ complex (see Fig. S2) have been deposited in the Research Collaboratory for Structural Bioinformatics Protein Data Bank with the code 9BI5. Cryo-EM density maps have been deposited in the Electron Microscopy Data bank with accession code EMD-44559.

## LIMITATIONS OF THE STUDY.

*In vivo*, the *S. cerevisiae* Mre11 complex consists of Mre11, Rad50 and Xrs2. We were unable to obtain particles of MRX for our studies. This restricted our structure-guided genetic analyses to Mre11 and Rad50. Also, we only got particles for MR^EQ^ and not for MR^WT^, thus preventing us to get structural insights of the complex after ATP-hydrolysis. In addition, the flexibility of the extended Rad50 coiled coil domain precluded us from obtaining structural information regarding suppressor mutations that reside within the coils, thus limiting our ability to determine the mechanism(s) of suppression. The molecular defect of *rad50–3KA* was mostly informed from Molecular Dynamics simulations and future studies are required to test the suggested model.
